# Vimentin prevents a miR-dependent negative regulation of tissue factor mRNA during epithelial–mesenchymal transitions and facilitates early metastasis

**DOI:** 10.1038/s41388-020-1244-1

**Published:** 2020-03-10

**Authors:** Marie-Emilie Francart, Aline M. Vanwynsberghe, Justine Lambert, Morgane Bourcy, Anthony Genna, Julien Ancel, Jennifer Perez-Boza, Agnès Noël, Philippe Birembaut, Ingrid Struman, Myriam Polette, Christine Gilles

**Affiliations:** 1 GIGA-Cancer, Laboratory of Tumor and Development Biology, University of Liège, Liège, Belgium; 2 Hôpital Maison Blanche, Service de pneumologie, CHU de Reims, 51092 Reims, France; 3 INSERM, P3Cell UMR-S1250, SFR CAP-SANTE, Université de Reims Champagne-Ardenne, 51097 Reims, France; 4 GIGA-Cancer, Molecular Angiogenesis Laboratory, University of Liège, Liège, Belgium; 5 Hôpital Maison Blanche, Laboratoire de pathologie, CHU de Reims, 51092 Reims, France

**Keywords:** Cell biology, Cancer

## Abstract

Epithelial–mesenchymal transitions (EMTs) are high-profile in the field of circulating tumor cells (CTCs). EMT-shifted CTCs are considered to encompass pre-metastatic subpopulations though underlying molecular mechanisms remain elusive. Our previous work identified tissue factor (TF) as an EMT-induced gene providing tumor cells with coagulant properties and supporting metastatic colonization by CTCs. We here report that vimentin, the type III intermediate filament considered a canonical EMT marker, contributes to TF regulation and positively supports coagulant properties and early metastasis. Different evidence further pointed to a new post-transcriptional regulatory mechanism of TF mRNA by vimentin: (1) vimentin silencing accelerated TF mRNA decay after actinomycin D treatment, reflecting TF mRNA stabilization, (2) RNA immunoprecipitation revealed enriched levels of TF mRNA in vimentin immunoprecipitate, (3) TF 3′-UTR-luciferase reporter vector assays implicated the 3′-UTR of TF mRNA in vimentin-dependent TF regulation, and (4) using different TF 3′UTR-luciferase reporter vectors mutated for potential miR binding sites and specific Target Site Blockers identified a key miR binding site in vimentin-dependent TF mRNA regulation. All together, these data support a novel mechanism by which vimentin interferes with a miR-dependent negative regulation of TF mRNA, thereby promoting coagulant activity and early metastasis of vimentin-expressing CTCs.

## Introduction

Circulating tumor cells (CTCs) have attracted enormous attention for their potential clinical significance [1, 2] (1) to help predicting metastases, (2) to help guiding treatment decisions and assessing therapeutic efficacy and (3) to allow a live monitoring of disease progression and recurrence. Containing metastatic founders, CTCs are thus recognized to harbor important potential not only as biomarkers but also as therapeutic cellular targets [3]. Epithelial-mesenchymal transitions (EMTs), providing tumor cells with enhanced invasive, survival, stemness, and niching properties, have rapidly been recognized key processes in the biology of CTCs liberated from epithelial tumors [4–7]. Accordingly, the expression of EMT actors (e.g., vimentin, Twist, Snail, or ZEB1) has been detected in CTCs in animal models and extensively reported in subpopulations of CTCs isolated from cancer patients [4, 7–9]. It is thus today considered that EMT-shifted CTCs encompass subpopulations of metastasis initiating cells. Nevertheless, the mechanisms underlying metastatic colonization by EMT-shifted CTCs remain elusive.

Several pieces of work support a contribution of coagulation in the process. Hypercoagulability is actually a long known correlate of malignancy and venous thromboembolism has been associated with worse prognosis [10–12]. In particular, CTCs have recently been associated with increased risk of venous thrombosis in cancer patients [13–16]. Though cancer-associated thrombosis is clearly multifactorial, an aberrant thrombotic activity of tumor cells is considered a key factor. Tissue factor (TF), a 47 kDa membrane-associated glycoprotein known as a potent membrane-associated activator of the coagulation cascade, has emerged as a central player in the relationship between the hemostatic system and cancer progression [11, 12, 17–20]. TF binds FVIIa and subsequentely triggers the downstream coagulation cascade leading to thrombin generation and fibrin-rich-clot formation. As we contributed to show [21], enhanced TF expression has been observed in several tumor cell lines and several types of human cancers [14, 22–27]. If TF also triggers cellular signaling events that facilitate tumor progression [12, 28, 29], a determinant role of TF-associated coagulation mechanisms in supporting metastasis has been demonstrated [10, 12, 17, 30, 31]. Notwithstanding the implication of TF-bearing tumor-derived microparticles in hypercoagulability, a local activation of coagulation is more particularly considered to contribute to the creation of a pericellular fibrin/platelet-rich cocoon protecting CTCs against shear stress, anoikis and immune attack and also providing a favorable niche for their early metastatic seeding [10, 12, 13, 17, 30, 32].

Recently, we identified an EMT-driven axis leading to the overexpression of TF, and providing tumor cells with coagulant properties that facilitate early metastatic colonization of CTCs in experimental metastasis mice assays [21]. Based on this previous work emphasizing a narrow association between vimentin and TF expression in vitro, in human breast cancers and in CTCs isolated from metastatic breast cancer patients, we examined here the possibility that the canonical EMT marker vimentin could directly contribute to TF regulation, and thereby to early metastasis. Vimentin is a type III intermediate filament which, in a normal and adult context, is mostly expressed by cells of mesenchymal origin [33]. It is today considered a canonical marker of both physiological and pathological EMTs. We and others have extensively reported vimentin expression in tumor cells of epithelial origin in vitro, in animal models, in a large variety of epithelial human tumors and CTCs isolated from cancer patients in which it associates with poor clinical parameters [4, 6, 21, 34–38]. As part of the cytoskeleton, vimentin has been primarily recognized as a structural protein but has later been functionally involved in various processes including cell migration and invasion, cell–cell or cell–substrate adhesion, resistance to shear stress and anoikis or drug resistance and metastasis [33, 39–46]. More particularly, vimentin has also been implicated in signalization and gene regulation [47–50].

In this study, we report a functional role of vimentin in regulating TF expression at a posttranscriptional level. We show, in several EMT cellular systems, that vimentin silencing diminishes TF expression, coagulant activity and early metastasis. Digging further the mechanisms underlying this regulation, vimentin was found to counteract a miR-dependent negative regulation of TF mRNA.

## Results

### Vimentin regulates TF expression and coagulant properties of EMT+ tumor cells

We recently reported that TF expression is induced by EMT, providing EMT-shifted cells with coagulant properties that facilitate early metastasis [21]. By immunohistochemistry, TF expression was more particularly shown to correlate with vimentin expression in in vitro models of EMT, in human breast cancers and in CTCs isolated from metastatic breast cancer patients [21]. We bring here further support to this narrow relationship linking TF and vimentin by exploring RNA expression public databases that revealed a positive correlation between the two molecules both on nonmetastatic and metastatic breast cancers (Supplementary Fig. 1a, b). Similar findings were generated by immunohistochemistry on metastatic and nonmetastatic lung adenocarcinomas (Supplementary Fig. 1c–e, Supplementary material and methods), suggesting that TF/vimentin association is an early cancer marker.

**Fig. 1:**
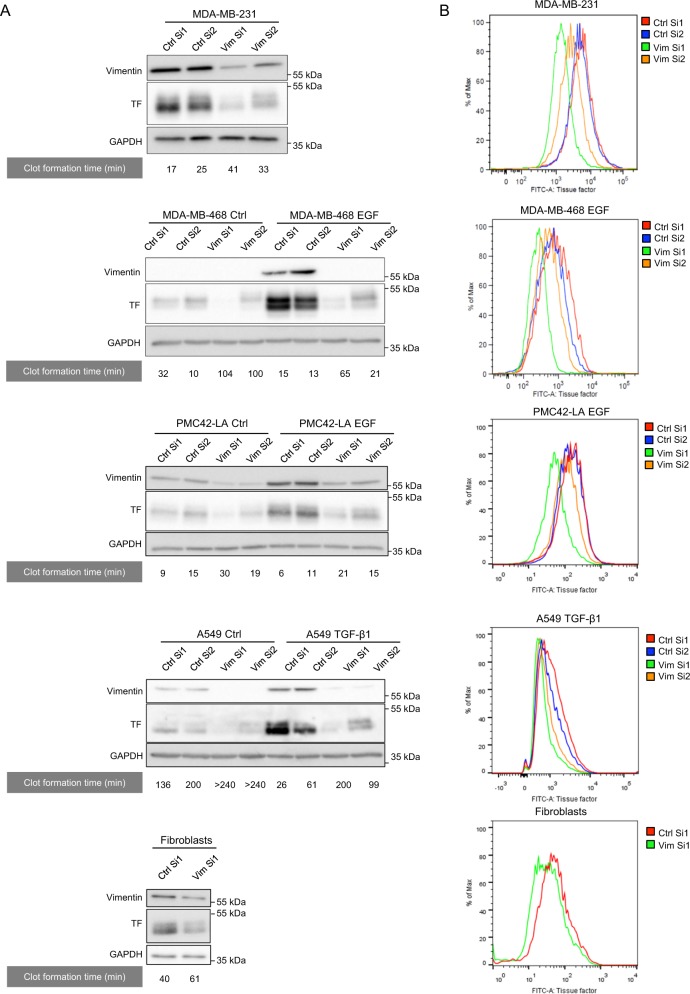
Impact of vimentin on TF expression and coagulant properties of EMT+ tumor cells. **a** Western blotting analyses of TF and GAPDH in MDA-MB-231, in MDA-MB-468 cells induced to EMT by EGF, in PMC42-LA cells induced to EMT by EGF, in human lung tumor A549 cells induced to EMT by TGF-β1*,* and human skin fibroblasts, and transfected with two nontargeting siRNA (Ctrl Si1 or Ctrl Si2) or two siRNA against vimentin (Vim Si1 or Vim Si2). The results of corresponding in vitro coagulation assays, performed by incubating whole blood of healthy donors with cells transfected, are given underneath the western blots. **b** FACS analyses of surface TF expression in cells treated as in **a**.

Considering this tight association between TF and vimentin expression and aiming at deciphering EMT-driven molecular mechanisms implicated in the regulation of TF expression, we here examined the potential contribution of vimentin to the process. For that purpose, we transfected siRNA sequences against vimentin (Vim Si1 and Vim Si2) in well-known EMT+ MDA-MB-231 human breast tumor cells and in human cell systems previously reported to be inducible for EMT by EGF (MDA-MB-468, PMC42-LA) or TGF-β1 (Α549). Silencing vimentin clearly inhibited TF expression in all cell models as shown by western blotting (Fig. 1a). We verified that downregulating vimentin did not modulate other cytoskeleton protein levels such as keratins, tubulin, or actin (Supplementary Fig. 2). Because cell-surface TF is determinant for the initiation of coagulation, we also showed by FACS that cell surface-associated TF expression is similarly decreased following vimentin silencing in our tumoral cell systems (Fig. 1b). Mean fluorescence intensity are provided as supplemental information (Supplementary Table 1).

We next evaluated whether vimentin-dependent modulation of TF expression functionally impacts cell coagulant activity. Using an in vitro clot formation assay, we showed that control cells are able to form a clot faster than cells inhibited for vimentin expression (Fig. 1a). It is noteworthy that Vim Si1, which is more efficient than Vim Si2 in inhibiting vimentin expression, correlatively better inhibited TF expression and coagulant properties (Fig. 1). Interestingly, similar observations were also made on human skin fibroblasts, supporting the existence of a vimentin/TF relationship in a normal cellular context (Fig. 1).

### Vimentin silencing hinders metastatic colonization

**Fig. 2:**
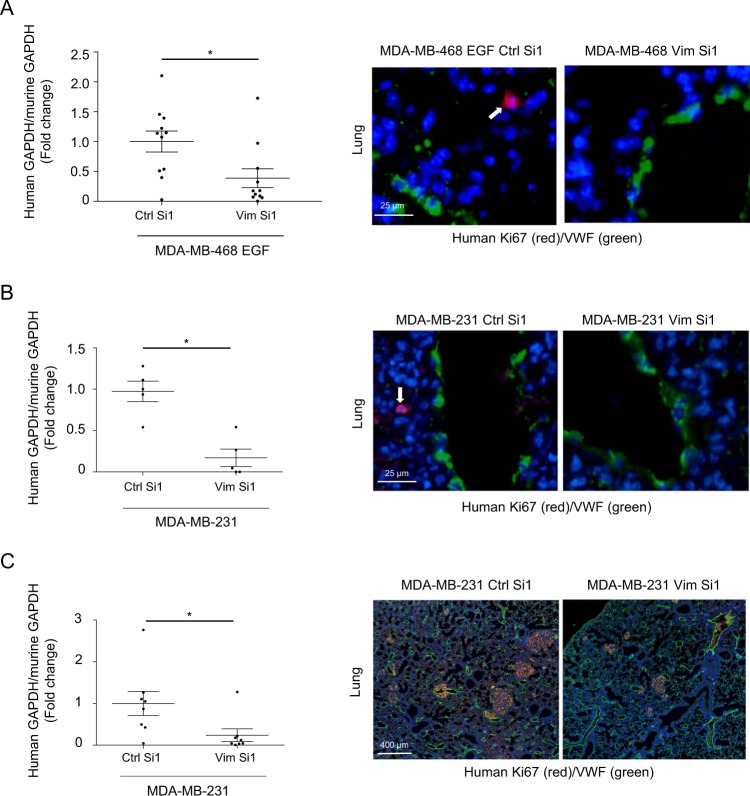
Impact of vimentin silencing on metastatic colonization. RT-nested qPCR for human GAPDH performed on total RNA extracted from lungs of BALB/c mice injected intravenously with EGF-treated MDA-MB-468 cells (**a**) (*n* = 11) or MDA-MB-231 (**b**) (*n* = 5) transfected with a non-targeting control siRNA (Ctrl Si1) or a siRNA against vimentin (Vim Si1) and collected 24 h after injection. **c** RT-nested qPCR for human GAPDH performed on total RNA extracted from lungs of SCID mice injected intravenously with MDA-MB-231 silenced for vimentin or not and sacrificed 3 weeks after injections (*n* = 8). Double immunofluorescence against human Ki67 (red) and mouse VWF (green) performed on lung sections. Nuclei were labeled with DAPI (blue).

Because TF expression has been shown by others and us to support early steps of metastatic colonization (survival and early niching), we examined the impact of silencing vimentin in short-term experimental metastasis models optimized previously in the laboratory [21]. In a scientific context suggesting that EMT supports early metastasis while MET must occur for metastasis to grow, we optimized these assays using cells transiently silenced in vitro before injection aiming at preferentially affecting early steps of metastasis. Comparing EGF-treated MDA-MB-468 (Fig. 2a) and MDA-MB-231 (Fig. 2b) cells transfected with Vim Si1 in vitro before injection, we observed a clear diminution of human tumor cell content in lungs after vimentin silencing, as quantified by RT-qPCR. Immunostaining for human Ki67 corroborated the presence of tumor cells in the lung parenchyma.

To confirm the ability of seeded cells to develop metastases, we examined the impact of TF regulation by vimentin on overall long-term metastasis formation. MDA-MB-231 cells silenced or not for vimentin were thus intravenously injected in SCID mice for 3 weeks to allow metastatic growth (Fig. 2c). Immunofluorescence against human Ki67 confirmed the presence of developed lung metastases in these long-term metastasis assays. Quantification revealed that mice injected with control cells displayed a higher level of human GAPDH in the collected lungs compared with mice injected with cells silenced for vimentin.

### Vimentin stabilizes TF mRNA

**Fig. 3:**
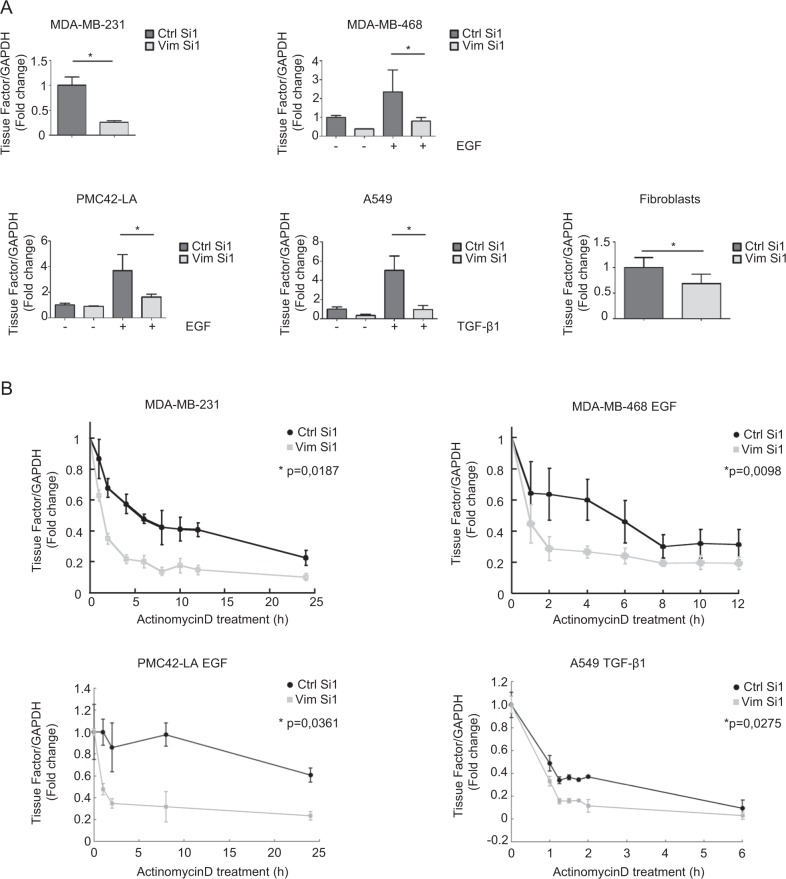
Stabilization of TF mRNA by vimentin. **a** RT-qPCR analyses of TF in various cellular systems transfected with a nontargeting control siRNA (Ctrl Si1) or an siRNA against vimentin (Vim Si1). **b** RT-qPCR analyses of TF in MDA-MB-231, EGF-treated MDA-MB-468, EGF-treated PMC42-LA and TGF-β1-treated A549 cells transfected with a vimentin siRNA (Vim Si1) or a control siRNA (Ctrl Si1) for 24 h and subsequently treated with actinomycin D for different time periods.

In the light of the clear regulation of TF by vimentin, we explored further the molecular mechanism underlying this regulation. We first observed that vimentin silencing decreased TF mRNA level in all cellular systems examined (Fig. 3a). Most importantly, silencing vimentin in MDA-MB-231 cells or EMT-induced cells (MDA-MB-468, A549 and PMC42-LA) was found to increase the decay of TF mRNA after actinomycin D treatment (Fig. 3b), suggesting that vimentin contributes to stabilize TF mRNA.

### Vimentin regulates TF mRNA 3′-UTR

**Fig. 4:**
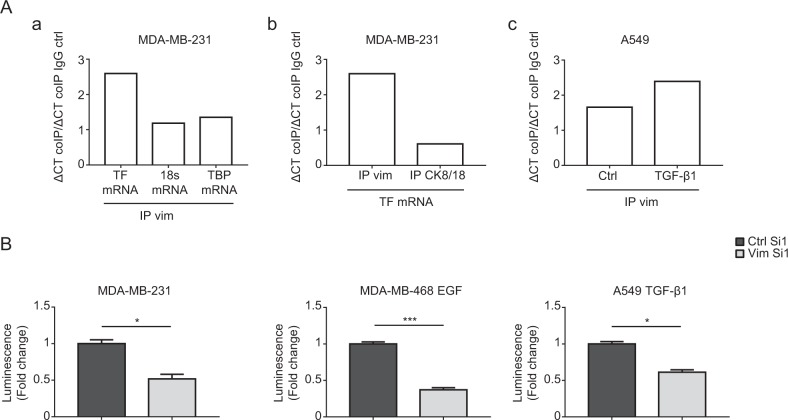
Regulation of TF mRNA 3′-UTR by vimentin. **a** RNA-immunoprecipitation assays. a MDA-MB-231 cell lysates were subjected to immunoprecipitation with a vimentin antibody (IP Vim). RNA was extracted from the immunoprecipitate and subjected to RT-qPCR for TF, 18S or TBP. b MDA-MB-231 cell lysates were subjected to immunoprecipitation with a vimentin (IP Vim) or a cytokeratin 8/18 antibody (IP CK8/18) and extracted RNAs were subjected to RT-qPCR for TF. Results from a representative experiment are shown. c Cell lysates from Ctrl or TGF-β1-treated A549 cells were subjected to immunoprecipitation with a vimentin antibody (IP Vim). RNA was extracted from the immunoprecipitate and subjected to RT-qPCR for TF. Immunoprecipitation conditions with control IgG were included in all experiments. **b** TF 3′-UTR-luciferase reporter assays. MDA-MB-231, EGF-treated MDA-MB-468 and TGF-β1-treated A549 cells were transfected with a reporter vector containing the firefly luciferase coding sequence inserted upstream the 3′-UTR of TF mRNA. The vector also expresses the renilla luciferase as an internal control. Results are expressed as the ratio firefly luciferase/renilla luciferase.

In order to determine whether vimentin could interact with TF mRNA, we took advantage of the MDA-MB-231 cells, which express high levels of both vimentin and TF to perform RNA immunoprecipitation (RIP) analyses. We observed that TF mRNA is enriched in a cellular protein fraction immunoprecipitated with a vimentin antibody, while other RNAs (18S or TBP) (Fig. 4a, panel a), examined as controls, are not enriched. This enrichment of TF mRNA also appeared to be specific to vimentin since it was not observed when analyzing a cytokeratin 8/18 immunoprecipitate (Fig. 4a, panel b). An enrichment of TF mRNA in the vimentin immunoprecipitate was also observable in TGF-β1-induced A549 cells (Fig. 4a, panel c). These data suggest that vimentin may, directly or indirectly, complex TF mRNA and increase its stability.

Supportively, a binding of vimentin to the 3′-UTR of specific mRNAs, enhancing their stability, has been reported in the literature [51]. To evaluate whether such a mechanism could similarly intervene on TF mRNA, we employed a TF 3′-UTR reporter vector containing the firefly luciferase coding sequence located upstream of the 3′-UTR of the TF mRNA. Transfecting this construct in MDA-MB-231, EGF-treated MDA-MB-468 and TGF-β1-treated A549 cells silenced for vimentin revealed a diminution of luciferase activity (Fig. 4b). These data taken together suggest the existence of a mechanism by which vimentin impairs a negative regulatory mechanism occurring in the 3′-UTR region of the TF mRNA.

### Vimentin interferes with miR-dependent TF mRNA regulation

**Fig. 5:**
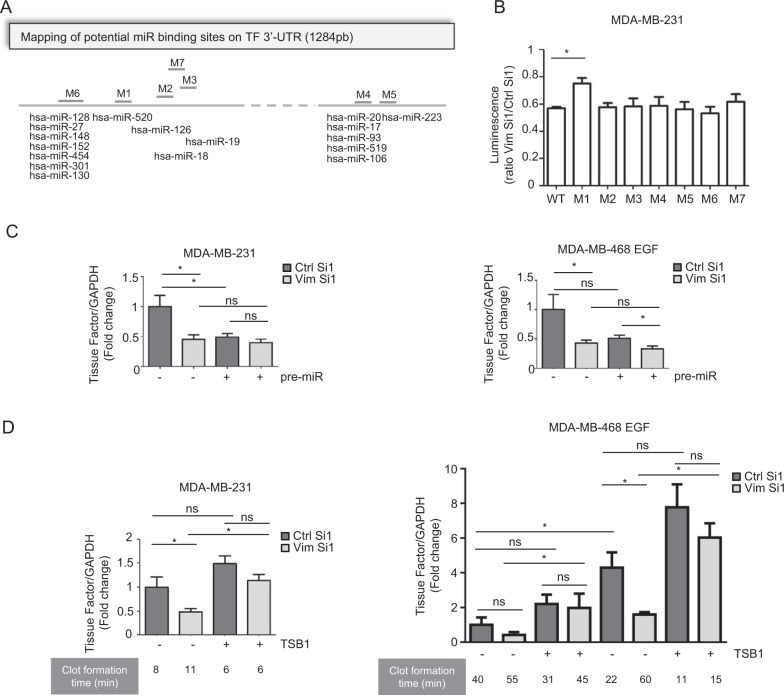
Modulation of miR-dependent TF mRNA regulation by vimentin. **a** Schematic representation of potential miR binding sites on the 3′-UTR of TF. Seven putative miR binding regions have been identified by TargetScan and literature search, and have been mutated (designated M1 to M7 on the map). **b** Luciferase reporter assays using the TF 3′-UTR reporter vectors mutated for the different binding sites of potentially interacting miRs. Results are expressed as a ratio between the luciferase activity in the Vim Si1 condition and in the Ctrl Si1 condition. **c** RT-qPCR analyses of TF in MDA-MB-231 or EGF-treated MDA-MB-468 cells co-transfected with a vimentin siRNA (Vim Si1) or a control siRNA (Ctrl Si1) and pre-miR-520g or a control pre-miR. **d** RT-qPCR analyses of TF and coagulation assays performed on MDA-MB-231 or MDA-MB-468 cells treated or not with EGF, and co-transfected with a vimentin siRNA (Vim Si1) or a control siRNA (Ctrl Si1) and a TSB1 encompassing the M1 region or a TSB control.

In line with this concept, several authors reported a negative regulation of TF mRNA at its 3′-UTR through miRNA-dependent mechanisms, both in tumoral and nontumoral cellular backgrounds [52–59]. We thus further examined whether vimentin could interfere with a miRNA-dependent regulation of TF. Combining a TargetScan [60] search (Version 7.1) with literature data, we identified 17 miRs that could potentially target 7 regions in the 3′-UTR of TF mRNA (Fig. 5a). We then generated several 3′-UTR-luciferase reporter vectors mutated at each of the potential binding sites of these miRNAs and evaluated the effect of vimentin silencing on the expression of these constructs in MDA-MB-231 cells. In this assay, the M1 construct was the only one for which the activity was less affected by vimentin silencing (Fig. 5b).

The M1 construct harbors a mutation in a potential binding site for several members of the miR-520 family, as validated for miR-520g by D’Asti et al. in human medulloblastoma cell lines [55]. In addition, we found that major members of the miR-520 family are expressed in our cellular models and we observed that their abundance is not dramatically modulated neither by EMT induction nor by vimentin silencing (Supplementary Fig. 3). We also showed that increasing miR-520g cellular content by transfecting pre-miR-520g in MDA-MB-231 cells or in the EMT-induced MDA-MB-468 cells decreased TF mRNA to a level that nevertheless did not drop further down after vimentin silencing (Fig. 5c). As predicted by miRDB [61], the M1 site of TF 3′-UTR is also a potential target of other miRs and we therefore cannot exclude the involvement of other miRs.

Considering this diversity and potential redundancy of miRs regulating TF, we rather aimed at interfering directly at the level of the M1 binding site in the TF mRNA. We thus used a Target Site Blocker (TSB1) designed to target the M1 sequence, thereby inhibiting miR binding. We showed that, both in MDA-MB-231 and in MDA-MB-468 cell models, TSB1 hindered the decrease of TF mRNA induced by vimentin silencing. Notably, TSB1 restored higher coagulant activity in the vimentin-silenced cells, demonstrating a functional role of this vimentin/miR/TF regulatory axis (Fig. 5d).

This set of data thus specifies a role for vimentin in preventing a miR-dependent negative regulation of TF mRNA. The M1 region in the TF 3′-UTR plays a key role in mediating the impact of vimentin on TF mRNA expression and on cell coagulant activity.

## Discussion

We here report a novel EMT-driven regulatory mechanism of TF expression in tumor cells by which vimentin protects TF mRNA from a miR-dependent downregulation. Our results further support a functional contribution of this vimentin-TF regulatory axis in providing tumor cells with enhanced coagulant properties and an increased competence to accomplish early metastasis.

If molecular relationships linking EMT and TF expression remain underexplored, diverse soluble factors and transcription factors, with recognized implication for EMT, have been shown to regulate TF in different cellular contexts. Thus, TF expression is induced both in normal cells (mostly vascular or fibroblastic-type cells and monocytes after injury or infection) and in tumor cells by a variety of inflammatory and growth factors including VEGF, TNFα, IL-1β, IL-6, or IL-8 [20]. Oncogenic pathways such as EGFR, c-Met, KRAS, loss of PTEN, or P53 also contribute to regulate TF expression [12, 20, 62, 63]. More directly linking EMT and TF expression and in line with our observations, Pr. Rak’s laboratory previously reported a modulation of TF expression in tumor cells by EGFR activation and E-cadherin blockade [64]. Our previous work additionally evidenced a prominent role of EMT transcription factors ZEB1 and Snail in the regulation of TF expression in tumor cells [21]. Considering the important, though not complete, phenotypic and molecular overlaps identified between EMT-derived cells and cancer stem cells (CSC), it is important to note that TF expression has also been associated to CSC markers, and that TF has recently been examined as a target for strategies aiming at eradicating tumor cells and CSCs in tumors [25–27, 65, 66].

The present work, identifying the canonical EMT marker vimentin as a regulator of TF expression, further strengthens this narrow relationship between EMT and TF expression. Increasing data indeed today show that, more than being a structural protein of the cytoskeleton, vimentin may also directly regulate gene expression. A signaling role of vimentin has thus been demonstrated, involving direct protein–protein interactions with molecular intermediates of signaling pathways [47, 48]. In direct support of our observations, vimentin has also been shown to bind and stabilize specific mRNAs including collagen α1 (I) and α2 (I) mRNA in fibroblasts, osteoblastic alkaline phosphatase mRNA in endothelial cells, the opioid receptor mRNA in neuroblastoma cells, or the heme-regulated inhibitor mRNA in leukemia cells [51, 67–69], though the precise mechanisms involved in these posttranscriptional regulations remain unclear. We here report an original mechanism supporting that vimentin stabilizes TF mRNA and interferes with a miR-dependent-negative regulation at its 3′-UTR. Accordingly, a regulation of TF mRNA by several specific miRs has previously been reported [58, 59]. For instance, miR-126, miR-19a, and miR-223 were shown to decrease TF levels in endothelial cells in a context of diabetes or inflammation [52, 53, 70], and miR-20b was found to regulate TF in embryonic stem cells [54] or in a context of lupus erythematosus [71]. Few studies also reported a miR-dependent regulation TF in cancer contexts [55–57]. Thus, miR-19 was identified as a miR highly expressed in poorly invasive MCF-7 breast tumor cells that contributes to maintain low level of TF in these cells when compared to invasive MDA-MB-231 expressing low levels of miR-19 and higher levels of TF [56]. In line with these observations, we observed, in the context of vimentin/EMT-positive MDA-MB-231 cells, that a mutation in the potential binding site of miR-19 (corresponding to our M3 mutant) in the TF 3′-UTR reporter vector does not hinder the diminution of luciferase activity observed after vimentin silencing. Our results indeed rather pointed to the M1 site as being implicated in the vimentin/miR-dependent regulation of TF mRNA. Accordingly, D’Asti et al., working with human medulloblastoma cell lines, previously identified this site as mediating a negative regulation of a TF 3′-UTR reporter by miR-520g [55]. The authors further showed that exogenous miR-520g downregulates TF levels and coagulant activity in the medulloblastoma model. We similarly observed an effect of exogenous pre-miR-520g on TF expression in the breast tumor cell context. Nevertheless, as predicted by miRDB, the M1 site of TF 3′-UTR is also a potential target of other miRs (i.e. miR-3609 and several miR-548 family members) and we cannot exclude a contribution of other miRs in the vimentin/TF regulation axis. Regardless of the nature of miRs involved, our observation that a TSB encompassing the M1 binding site hinders the downregulation of TF mRNA induced by vimentin silencing thus confirmed the results obtained with the M1 mutant 3′-UTR reporter and corroborated the existence of a mechanism by which vimentin interferes with a miR-dependent negative regulation of TF.

In addition, our results support a functional role of this vimentin/TF regulatory axis in providing tumor cells with enhanced coagulant activity and increased metastatic colonization abilities. Silencing vimentin indeed inhibited the coagulant properties of tumor cells and co-transfecting the TSB1 attenuated this effect. Vimentin silencing also decreased the ability of tumor cells to accomplish metastatic colonization when injected as CTCs in experimental metastasis assays. These data bridge two independent sets of literature data including ours supporting, on one hand, that EMT-shifted CTCs represent a subpopulation of CTCs with enhanced metastatic competence and, on the other hand, that TF-dependent coagulant properties of CTCs facilitate their survival in the blood stream and metastatic colonization [10–12, 30]. Accordingly, we previously reported the existence of CTCs co-expressing vimentin and TF in the blood of breast cancer patients [21].

All together, these data support the existence of a mechanism by which vimentin can protect TF mRNA by interfering with a miR-dependent negative regulation mechanism of TF mRNA. Such a mechanism could contribute to equip EMT + CTCs expressing vimentin with higher coagulant properties, thereby facilitating early colonization processes.

## Material and methods

### Cell culture

Human breast MDA-MB-468 cancer cell line was obtained from the ATCC (Manassas, VA). MDA-MB-231 and A549 luciferase-expressing clones were purchased from Caliper Life Sciences (Waltham, MA). The breast cancer PMC42-LA subline was obtained from Dr. M.L. Ackland (Deakin University, Burwood, Australia) [72]. All cell lines were used within ten passages after authentication (STR DNA typing, Leibniz-Institute DSMZ), and were mycoplasma free. Cells were cultured in DMEM (Gibco, Thermo Fisher Scientific, Waltham, MA) supplemented with 10% FBS or in RPMI (Gibco, Thermo Fisher Scientific) supplemented with 10% FBS for PMC42-LA. For EMT induction, inducible cell lines were treated for 48 h with 20 ng/ml recombinant EGF (Sigma-Aldrich, Saint-Louis, MO) or 5 ng/ml recombinant TGF-β1 (R&D Systems, Minneapolis, MN).

Human skin fibroblasts were isolated from normal human dermis explants and amplified in DMEM supplemented with 7% FBS. These cells were kindly provided by Drs Alain Colige and Charles Lambert (Laboratory of Connective Tissue Biology, University of Liège, Belgium).

### RT-qPCR, western blotting analyses, and flow cytometry detection of cell-surface TF

RT-qPCR was performed on RNA extracted from cell cultures with High Pure RNA Isolation Kit, reverse transcribed using the first-strand cDNA Synthesis kit and amplified on the LightCycler480 with the Universal Probe Library system (all kits are from Roche, Basel, Switzerland). Primer sequences are provided in Supplementary Table 2.

For western blotting analyses, total proteins were separated on 10% SDS-polyacrylamide electrophoresis gels and transferred to PVDF membranes. The antibodies used are listed in Supplementary Table 3.

For the detection of cell surface-associated TF, cells were detached with trypsin-EDTA, labeled with a FITC-conjugated monoclonal antibody against human TF (Supplementary Table 3) and analyzed with the FACS CantoII (GIGA-Imagery platform).

### siRNA transfection

Cells were transfected with RNAiMax (Invitrogen, Thermo Fisher Scientific) and 20 nM of the siRNA duplexes 24 h after plating. Specific 19-nt sequences were selected in the coding sequence of vimentin to generate 21-nt sense and 21-nt antisense strands of the type (19 N) TT (N, any nucleotide). The siRNA sequences were purchased from Eurogentec (Liège, Belgium) and are listed in Supplementary Table 4. Cells were harvested for subsequent analyses 48 h after transfection.

For actinomycin D (Sigma-Aldrich) experiments, cells were transfected with Vim Si1 or Ctrl Si1 24 h before the addition of 10 µg/ml actinomycin D in their culture media for different time periods (up to 24 h).

### Target site blockers (TSB) and pre-miR transfection

For TSB transfection experiments, a first transfection was performed for 6 h with RNAiMax and 20 or 50 nM of the TSB for MDA-MB-468 or MDA-MB-231 cells, respectively. Cells were then co-transfected for 18 h with RNAiMax and 20 or 50 nM of the TSB together with 20 nM of siRNA duplexes. TSB sequences were purchased from Qiagen (Hilden, Germany) and are listed in Supplementary Table 5. For pre-miR experiments, cells were co-transfected for 24 h with RNAiMax and 20 nM of the pre-miR together with 20 nM of siRNA duplexes. Pre-miR precursors were purchased from Ambion (PM10365 and AM17110, LifeTechnologies, Carlsbad, CA).

### RNA Immunoprecipitation

RIP was performed with the EZ-Magna RIP RNA-Binding Protein Immunoprecipitation Kit™ (Millipore, Burlington, MA) according to the instructions of the manufacturer. Ten micrograms of antibodies (listed in Supplementary Table 3) was used to precipitate proteins of interest. Control IgG were used as controls. RNA was extracted from the immunoprecipitate and analyzed by RT-qPCR for the mRNA of interests. Results are normalized to the IgG control condition.

### Dual-luciferase reporter assay

Cells transfected with vimentin siRNA as above were subsequently transfected using lipofectamine 2000 or LipoStem reagent (Invitrogen, Thermo Fisher Scientific) for 24 h with the reporter vector containing the firefly luciferase-coding sequence inserted upstream the 3′-UTR of TF mRNA. This vector also carries the renilla luciferase cDNA as an internal control (Genecopoeia Rockville, MD). The firefly and renilla luciferase activities were measured using the Dual-Luciferase^®^ Reporter (DLR™) Assay System (Promega, Madison, WI) according to the instructions of manufacturer. TF 3′-UTR vectors mutated for seven seeding regions were generated by Genscript (Nanjing, China). The sequences of the WT and the mutated TF 3′-UTR are listed in Supplementary Table 6.

### miRNA detection

Total RNA was extracted using the miRNeasy kit (Qiagen) following the manufacturer’s protocol. Fifty nanograms of RNA was reverse transcribed into cDNA using the qScript miRNA cDNA Synthesis kit (Quanta Biosciences, Beverly, MA), and RT-qPCR was conducted in triplicate using Perfecta SYBR Green Super Mix (Quanta Biosciences). Amplification was performed on an Applied Biosystems 7900 HT detection system (Applied Biosystems, Foster City, CA). The relative miRNA levels were normalized to two internal controls SNORD 44 and SNORD 48 using the delta–delta Ct method. Primers were purchased from Integrated DNA Technologies (Coralville, IA) (listed in Supplementary Table 7).

### Clotting assay

For the visual clotting assay, whole blood was collected from healthy donors on 3.2% sodium citrate. Forty-eight hours siRNA-transfected cells or TSB and siRNA co-transfected cells were suspended in 600 µl of serum-free DMEM (CaCl_2_ 1.2 mM) and exposed to 300 µl of blood. Clot formation time was monitored. All clotting experiments were performed at least three times during an observation period of 4 h. Due to interpersonal variability regarding clotting time, results from one representative experiment are shown.

### Mice models

All animal studies were approved by the Animal Ethics Committee of the University of Liège (no. 1932, ULiège, Belgium). BALB/c and SCID mice (7 weeks of age) were purchased from Charles River Laboratories (Wilmington, MA). After siRNA transfection, cells (1 × 10^5^ cells per mouse) were injected in the tail vein. The software G-power was used to determine the adequate sample size per group.

To quantify CTC persistence/early seeding, mice were sacrificed 24 h after intravenous (IV) injection. After tissue disruption in MagNa lyser Green beads tubes (Roche), total RNA was extracted from lungs with NucleoSpin RNA Midi kit (Macherey-Nagel, Düren, Germany). Human GAPDH levels were quantified by RT-nested qPCR, as previously described [21, 37], to evaluate tumor cell contents. In parallel, murine GAPDH was amplified from the same reverse-transcribed RNA using specific murine GAPDH primers. The sequences of the primers used are provided in Supplementary Table 1. Human GAPDH levels were normalized to the corresponding murine GAPDH levels for each mouse. The values are normalized to mean expression in the reference group in order to combine independent mice experiments.

For the long-term model, mice were sacrificed 3 weeks after IV injection and tumor content was evaluated in lungs by human/murine GAPDH RT-qPCR analyses as described above. Double immunofluorescence against Ki67 and Von Willebrand factor (VWF) to label blood vessels was also performed on paraffin-embedded mouse lungs as previously described [37] (see details for antibodies in Supplementary Table 3).

### Statistical analysis

Results are expressed as mean ± SEM (*n* = 3, for in vitro experiments). Statistical analyses were performed with Prism software (GraphPad software). In vitro results expressed as fold induction were analyzed with a two-tailed one-sample *t*-test. Actinomycin D results were analyzed with a Kolmogorov–Smirnov test. In vivo results were analyzed with a two-tailed Mann–Whitney test. A *P* < 0.05 was considered statistically significant. *P < 0.05, **P < 0.01, *** *P* < 0.001.

## Supplementary information

Supplementary Figures

Supplementary Tables

Supplementary Material and Methods

## References

[1] Cabel L, Proudhon C, Gortais H, Loirat D, Coussy F, Pierga JY (2017). Circulating tumor cells: clinical validity and utility. Int J Clin Oncol.

[2] Alix-Panabieres C, Pantel K (2016). Clinical applications of circulating tumor cells and circulating tumor DNA as liquid biopsy. Cancer Discov.

[3] Wang H, Stoecklein NH, Lin PP, Gires O (2017). Circulating and disseminated tumor cells: diagnostic tools and therapeutic targets in motion. Oncotarget.

[4] Francart ME, Lambert J, Vanwynsberghe AM, Thompson EW, Bourcy M, Polette M (2018). Epithelial-mesenchymal plasticity and circulating tumor cells: travel companions to metastases. Dev Dyn.

[5] Pastushenko I, Blanpain C (2019). EMT transition states during tumor progression and metastasis. Trends Cell Biol.

[6] Jolly MK, Somarelli JA, Sheth M, Biddle A, Tripathi SC, Armstrong AJ (2019). Hybrid epithelial/mesenchymal phenotypes promote metastasis and therapy resistance across carcinomas. Pharmacol Ther.

[7] Alix-Panabieres C, Mader S, Pantel K (2017). Epithelial-mesenchymal plasticity in circulating tumor cells. J Mol Med.

[8] Markiewicz A, Zaczek AJ (2017). The landscape of circulating tumor cell research in the context of epithelial-mesenchymal transition. Pathobiology.

[9] Yu M, Bardia A, Wittner BS, Stott SL, Smas ME, Ting DT (2013). Circulating breast tumor cells exhibit dynamic changes in epithelial and mesenchymal composition. Science.

[10] Ruf W, Rothmeier AS, Graf C (2016). Targeting clotting proteins in cancer therapy—progress and challenges. Thromb Res.

[11] Unlu B, Versteeg HH (2018). Cancer-associated thrombosis: the search for the holy grail continues. Res Pract Thromb Haemost.

[12] Hisada Y, Mackman N (2019). Tissue factor and cancer: regulation, tumor growth, and metastasis. Semin Thromb Hemost.

[13] Bystricky B, Reuben JM, Mego M (2017). Circulating tumor cells and coagulation-minireview. Crit Rev Oncol Hematol.

[14] Mitrugno A, Tormoen GW, Kuhn P, McCarty OJ (2016). The prothrombotic activity of cancer cells in the circulation. Blood Rev.

[15] Beinse G, Berger F, Cottu P, Dujaric ME, Kriegel I, Guilhaume MN (2017). Circulating tumor cell count and thrombosis in metastatic breast cancer. J Thromb Haemost.

[16] Mego M, De Giorgi U, Broglio K, Dawood S, Valero V, Andreopoulou E (2009). Circulating tumour cells are associated with increased risk of venous thromboembolism in metastatic breast cancer patients. Br J Cancer.

[17] Gil-Bernabe AM, Lucotti S, Muschel RJ (2013). Coagulation and metastasis: what does the experimental literature tell us?. Br J Haematol.

[18] Palumbo JS (2008). Mechanisms linking tumor cell-associated procoagulant function to tumor dissemination. Semin Thromb Hemost.

[19] Garnier D, Milsom C, Magnus N, Meehan B, Weitz J, Yu J (2010). Role of the tissue factor pathway in the biology of tumor initiating cells. Thromb Res.

[20] Ruf W, Riewald M. Regulation of tissue factor expression. In: Madame Curie Bioscience Database. Austin (TX): Landes Bioscience; 2013. https://www.ncbi.nlm.nih.gov/books/NBK6620/.

[21] Bourcy M, Suarez-Carmona M, Lambert J, Francart ME, Schroeder H, Delierneux C (2016). Tissue factor induced by epithelial-mesenchymal transition triggers a procoagulant state that drives metastasis of circulating tumor cells. Cancer Res.

[22] Zhang X, Li Q, Zhao H, Ma L, Meng T, Qian J (2017). Pathological expression of tissue factor confers promising antitumor response to a novel therapeutic antibody SC1 in triple negative breast cancer and pancreatic adenocarcinoma. Oncotarget.

[23] Zhao X, Cheng C, Gou J, Yi T, Qian Y, Du X (2018). Expression of tissue factor in human cervical carcinoma tissue. Exp Ther Med.

[24] Ueno T, Toi M, Koike M, Nakamura S, Tominaga T (2000). Tissue factor expression in breast cancer tissues: its correlation with prognosis and plasma concentration. Br J Cancer.

[25] Theunissen JW, Cai AG, Bhatti MM, Cooper AB, Avery AD, Dorfman R (2018). Treating tissue factor-positive cancers with antibody-drug conjugates that do not affect blood clotting. Mol Cancer Ther.

[26] Breij EC, de Goeij BE, Verploegen S, Schuurhuis DH, Amirkhosravi A, Francis J (2014). An antibody-drug conjugate that targets tissue factor exhibits potent therapeutic activity against a broad range of solid tumors. Cancer Res.

[27] Hu Z, Xu J, Cheng J, McMichael E, Yu L, Carson WE (2017). Targeting tissue factor as a novel therapeutic oncotarget for eradication of cancer stem cells isolated from tumor cell lines, tumor xenografts and patients of breast, lung and ovarian cancer. Oncotarget.

[28] Aberg M, Siegbahn A. Tissue factor non-coagulant signalling - molecular mechanisms and biological consequences with focus on cell migration and apoptosis. J Thromb Haemost. 2013;11:817–25. 10.1111/jth.12156 23384027

[29] Rothmeier AS, Liu E, Chakrabarty S, Disse J, Mueller BM, Ostergaard H (2018). Identification of the integrin-binding site on coagulation factor VIIa required for proangiogenic PAR2 signaling. Blood.

[30] Palumbo JS, Talmage KE, Massari JV, La Jeunesse CM, Flick MJ, Kombrinck KW (2007). Tumor cell-associated tissue factor and circulating hemostatic factors cooperate to increase metastatic potential through natural killer cell-dependent and-independent mechanisms. Blood.

[31] Liu S, Zhang Y, Zhao X, Wang J, Di C, Zhao Y, et al. Tumor-specific silencing of tissue factor suppresses metastasis and prevents cancer-associated hypercoagulability. Nano Lett. 2019;19:4721–30. 10.1021/acs.nanolett.9b01785 31180684

[32] Biggerstaff JP, Weidow B, Dexheimer J, Warnes G, Vidosh J, Patel S (2008). Soluble fibrin inhibits lymphocyte adherence and cytotoxicity against tumor cells: implications for cancer metastasis and immunotherapy. Clin Appl Thromb Hemost.

[33] Lowery J, Kuczmarski ER, Herrmann H, Goldman RD (2015). Intermediate filaments play a pivotal role in regulating cell architecture and function. J Biol Chem.

[34] Sarrio D, Rodriguez-Pinilla SM, Hardisson D, Cano A, Moreno-Bueno G, Palacios J (2008). Epithelial-mesenchymal transition in breast cancer relates to the basal-like phenotype. Cancer Res.

[35] Dauphin M, Barbe C, Lemaire S, Nawrocki-Raby B, Lagonotte E, Delepine G (2013). Vimentin expression predicts the occurrence of metastases in non small cell lung carcinomas. Lung Cancer.

[36] Yamashita N, Tokunaga E, Kitao H, Hisamatsu Y, Taketani K, Akiyoshi S (2013). Vimentin as a poor prognostic factor for triple-negative breast cancer. J Cancer Res Clin Oncol.

[37] Bonnomet A, Syne L, Brysse A, Feyereisen E, Thompson EW, Noel A (2012). A dynamic in vivo model of epithelial-to-mesenchymal transitions in circulating tumor cells and metastases of breast cancer. Oncogene.

[38] Satelli A, Batth IS, Brownlee Z, Rojas C, Meng QH, Kopetz S (2016). Potential role of nuclear PD-L1 expression in cell-surface vimentin positive circulating tumor cells as a prognostic marker in cancer patients. Sci Rep.

[39] Gilles C, Polette M, Zahm JM, Tournier JM, Volders L, Foidart JM (1999). Vimentin contributes to human mammary epithelial cell migration. J Cell Sci.

[40] Sun BO, Fang Y, Li Z, Chen Z, Xiang J (2015). Role of cellular cytoskeleton in epithelial-mesenchymal transition process during cancer progression. Biomed Rep.

[41] Sutoh Yoneyama M, Hatakeyama S, Habuchi T, Inoue T, Nakamura T, Funyu T (2014). Vimentin intermediate filament and plectin provide a scaffold for invadopodia, facilitating cancer cell invasion and extravasation for metastasis. Eur J Cell Biol.

[42] Matrone MA, Whipple RA, Balzer EM, Martin SS (2010). Microtentacles tip the balance of cytoskeletal forces in circulating tumor cells. Cancer Res.

[43] Wang W, Yi M, Zhang R, Li J, Chen S, Cai J (2018). Vimentin is a crucial target for anti-metastasis therapy of nasopharyngeal carcinoma. Mol Cell Biochem.

[44] Satelli A, Li S (2011). Vimentin in cancer and its potential as a molecular target for cancer therapy. Cell Mol Life Sci.

[45] Ivaska J (2011). Vimentin: Central hub in EMT induction?. Small GTPases.

[46] Dmello C, Sawant S, Alam H, Gangadaran P, Tiwari R, Dongre H (2016). Vimentin-mediated regulation of cell motility through modulation of beta4 integrin protein levels in oral tumor derived cells. Int J Biochem Cell Biol.

[47] Virtakoivu R, Mai A, Mattila E, De Franceschi N, Imanishi SY, Corthals G (2015). Vimentin-ERK signaling uncouples slug gene regulatory function. Cancer Res.

[48] Perlson E, Michaelevski I, Kowalsman N, Ben-Yaakov K, Shaked M, Seger R (2006). Vimentin binding to phosphorylated Erk sterically hinders enzymatic dephosphorylation of the kinase. J Mol Biol.

[49] Phua DC, Humbert PO, Hunziker W (2009). Vimentin regulates scribble activity by protecting it from proteasomal degradation. Mol Biol Cell.

[50] Satelli A, Hu J, Xia X, Li S (2016). Potential function of exogenous vimentin on the activation of Wnt signaling pathway in cancer cells. J Cancer.

[51] Schmidt Y, Biniossek M, Stark GB, Finkenzeller G, Simunovic F (2015). Osteoblastic alkaline phosphatase mRNA is stabilized by binding to vimentin intermediary filaments. Biol Chem.

[52] Li S, Chen H, Ren J, Geng Q, Song J, Lee C (2014). MicroRNA-223 inhibits tissue factor expression in vascular endothelial cells. Atherosclerosis.

[53] Witkowski M, Tabaraie T, Steffens D, Friebel J, Dorner A, Skurk C (2018). MicroRNA-19a contributes to the epigenetic regulation of tissue factor in diabetes. Cardiovasc Diabetol.

[54] Yu YH, Wu DS, Huang FF, Zhang Z, Liu LX, Zhang J (2013). MicroRNA-20b and ERK1/2 pathway independently regulate the expression of tissue factor in hematopoietic and trophoblastic differentiation of human embryonic stem cells. Stem Cell Res Ther.

[55] D’Asti E, Huang A, Kool M, Meehan B, Chan JA, Jabado N (2016). Tissue factor regulation by miR-520g in primitive neuronal brain tumor cells: a possible link between oncomirs and the vascular tumor microenvironment. Am J Pathol.

[56] Zhang X, Yu H, Lou JR, Zheng J, Zhu H, Popescu NI (2011). MicroRNA-19 (miR-19) regulates tissue factor expression in breast cancer cells. J Biol Chem.

[57] Yu G, Li H, Wang X, Wu T, Zhu J, Huang S (2013). MicroRNA-19a targets tissue factor to inhibit colon cancer cells migration and invasion. Mol Cell Biochem.

[58] D’Asti E, Rak J (2016). Biological basis of personalized anticoagulation in cancer: oncogene and oncomir networks as putative regulators of coagulopathy. Thromb Res.

[59] Eisenreich A, Leppert U (2014). The impact of microRNAs on the regulation of tissue factor biology. Trends Cardiovasc Med.

[60] Agarwal V, Bell GW, Nam JW, Bartel DP Predicting effective microRNA target sites in mammalian mRNAs. Elife 2015;4:e05005. 10.7554/eLife.05005 PMC4532895 26267216

[61] Wong N, Wang X (2015). miRDB: an online resource for microRNA target prediction and functional annotations. Nucleic Acids Res.

[62] Magnus N, D’Asti E, Meehan B, Garnier D, Rak J (2014). Oncogenes and the coagulation system – forces that modulate dormant and aggressive states in cancer. Thromb Res.

[63] Provencal M, Labbe D, Veitch R, Boivin D, Rivard GE, Sartelet H (2009). c-Met activation in medulloblastoma induces tissue factor expression and activity: effects on cell migration. Carcinogenesis.

[64] Milsom CC, Yu JL, Mackman N, Micallef J, Anderson GM, Guha A (2008). Tissue factor regulation by epidermal growth factor receptor and epithelial-to-mesenchymal transitions: effect on tumor initiation and angiogenesis. Cancer Res.

[65] de Bono JS, Concin N, Hong DS, Thistlethwaite FC, Machiels JP, Arkenau HT, et al. Tisotumab vedotin in patients with advanced or metastatic solid tumours (InnovaTV 201): a first-in-human, multicentre, phase 1-2 trial. Lancet Oncol. 2019;20:383–93. 10.1016/S1470-2045(18)30859-3 30745090

[66] Milsom C, Magnus N, Meehan B, Al-Nedawi K, Garnier D, Rak J (2009). Tissue factor and cancer stem cells: is there a linkage?. Arterioscler Thromb Vasc Biol.

[67] Aa Challa, B. Stefanovic (2011). A novel role of vimentin filaments: binding and stabilization of collagen mRNAs. Mol Cell Biol.

[68] Song KY, Choi HS, Law PY, Wei LN, Loh HH (2013). Vimentin interacts with the 5’-untranslated region of mouse mu opioid receptor (MOR) and is required for post-transcriptional regulation. RNA Biol.

[69] Chatterjee S, Panda AC, Berwal SK, Sreejith RK, Ritvika C, Seshadri V (2013). Vimentin is a component of a complex that binds to the 5’-UTR of human heme-regulated eIF2alpha kinase mRNA and regulates its translation. FEBS Lett.

[70] Witkowski M, Weithauser A, Tabaraie T, Steffens D, Krankel N, Witkowski M (2016). Micro-RNA-126 reduces the blood thrombogenicity in diabetes mellitus via targeting of tissue factor. Arterioscler Thromb Vasc Biol.

[71] Teruel R, Perez-Sanchez C, Corral J, Herranz MT, Perez-Andreu V, Saiz E (2011). Identification of miRNAs as potential modulators of tissue factor expression in patients with systemic lupus erythematosus and antiphospholipid syndrome. J Thromb Haemost.

[72] Ackland ML, Newgreen DF, Fridman M, Waltham MC, Arvanitis A, Minichiello J (2003). Epidermal growth factor-induced epithelio-mesenchymal transition in human breast carcinoma cells. Lab Investig.

