# Supplementary material for: Vimentin prevents a miR-dependent negative regulation of tissue factor mRNA during epithelial–mesenchymal transitions and facilitates early metastasis
Source: Oncogene. 2020 Mar 10;39(18):3680–92. doi: 10.1038/s41388-020-1244-1 (PMC7190572; doi:10.1038/s41388-020-1244-1)
Supplement: Supplementary file 1 — Supplementary Figures [file 41388_2020_1244_MOESM1_ESM.pptx]

## Slide 1
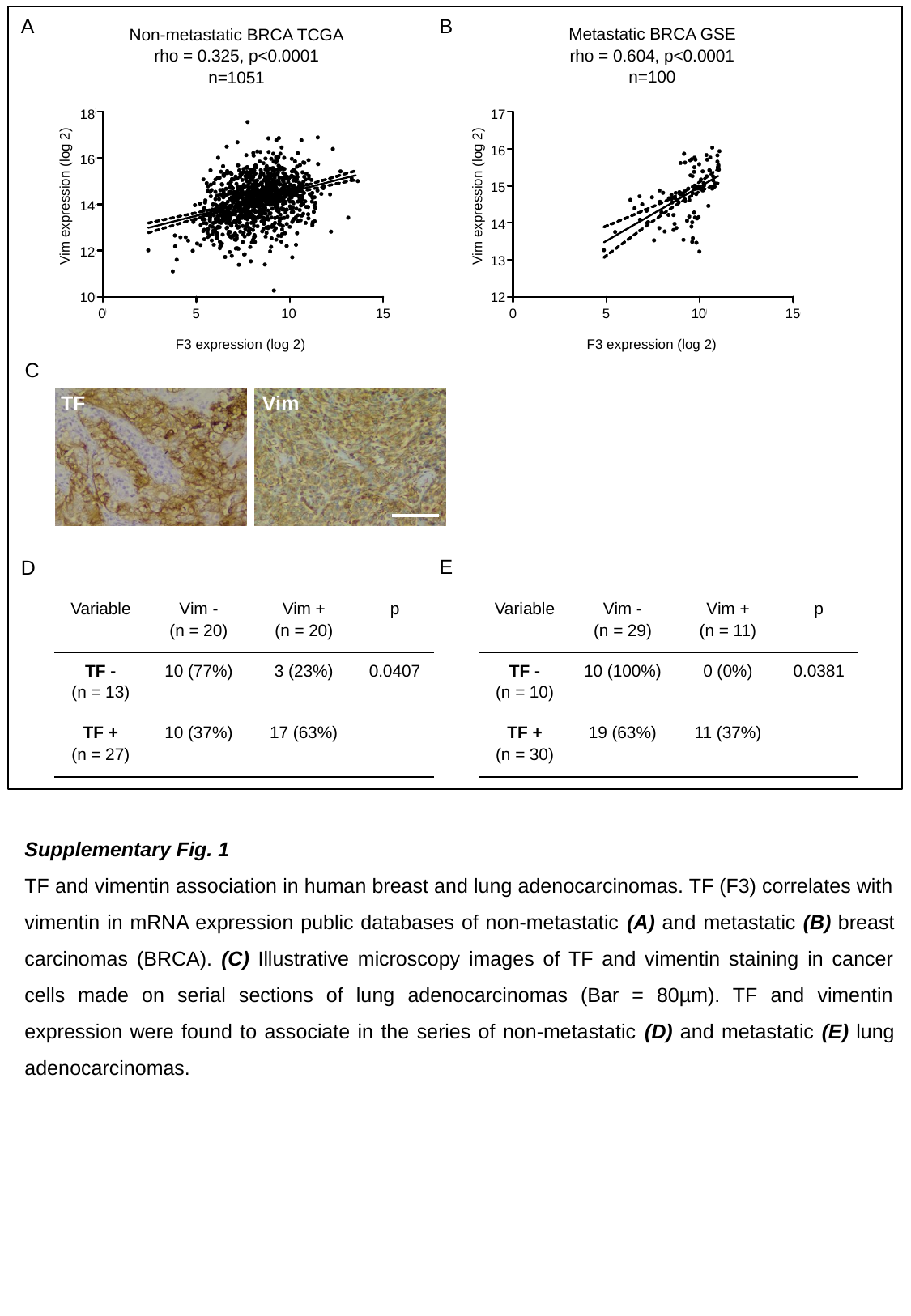

A
B
| Non-metastatic BRCA TCGA |
| --- |
| rho = 0.325, p<0.0001 |
| n=1051 |
| Metastatic BRCA GSE |
| --- |
| rho = 0.604, p<0.0001 |
| n=100 |
Vim expression (log 2)
Vim expression (log 2)
17
18
16
16
15
14
14
F3 expression (log 2)
F3 expression (log 2)
12
13
12
10
0
5
10
15
0
5
10
15
C
TF
Vim
E
D
| Variable | Vim - (n = 20) | Vim + (n = 20) | p |
| --- | --- | --- | --- |
| TF - (n = 13) | 10 (77%) | 3 (23%) | 0.0407 |
| TF + (n = 27) | 10 (37%) | 17 (63%) | |
| Variable | Vim - (n = 29) | Vim + (n = 11) | p |
| --- | --- | --- | --- |
| TF - (n = 10) | 10 (100%) | 0 (0%) | 0.0381 |
| TF + (n = 30) | 19 (63%) | 11 (37%) | |
Supplementary Fig. 1
TF and vimentin association in human breast and lung adenocarcinomas. TF (F3) correlates with vimentin in mRNA expression public databases of non-metastatic (A) and metastatic (B) breast carcinomas (BRCA). (C) Illustrative microscopy images of TF and vimentin staining in cancer cells made on serial sections of lung adenocarcinomas (Bar = 80µm). TF and vimentin expression were found to associate in the series of non-metastatic (D) and metastatic (E) lung adenocarcinomas.

## Slide 2
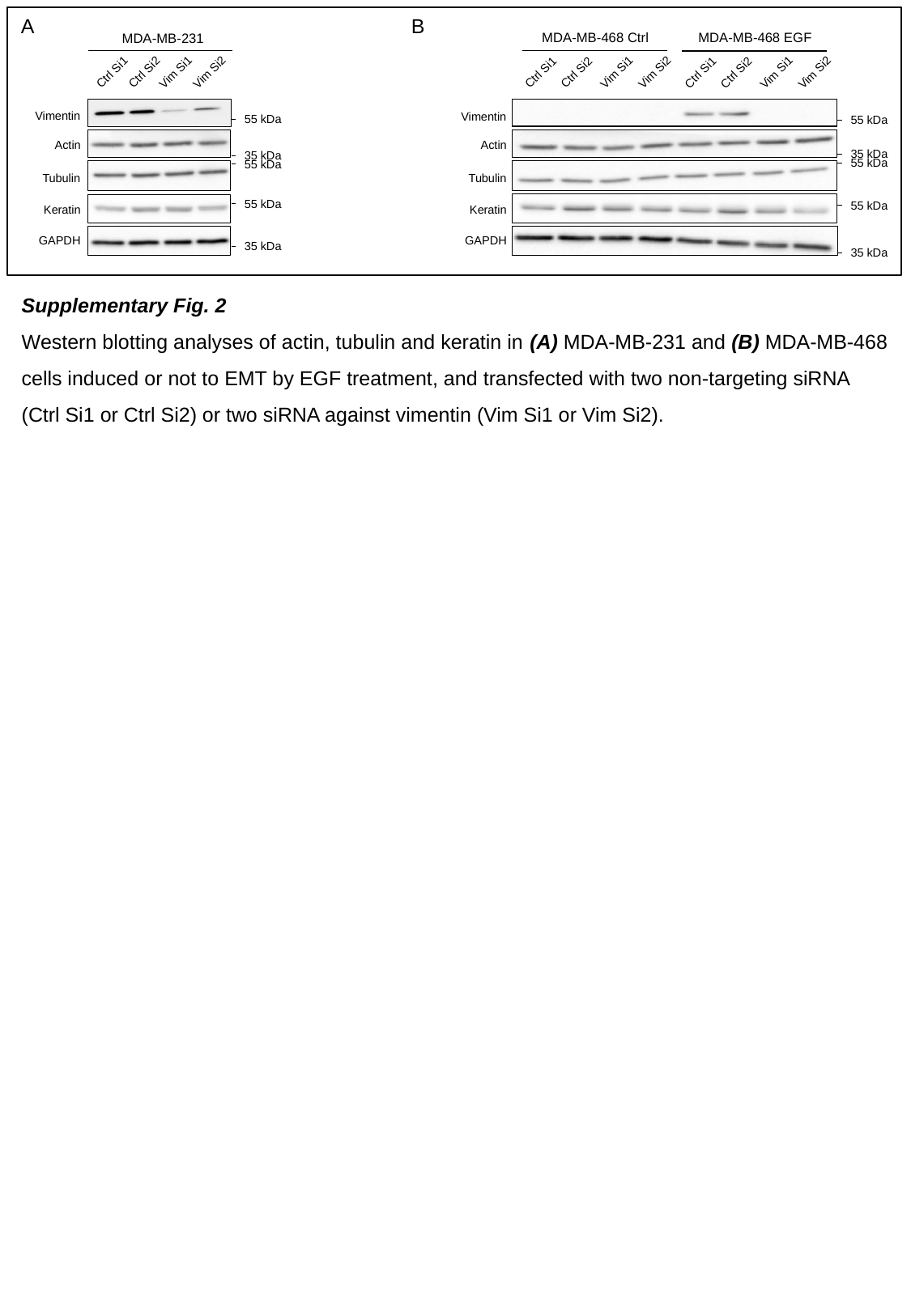

A
B
MDA-MB-468 Ctrl
MDA-MB-468 EGF
MDA-MB-231
Ctrl Si1
Ctrl Si2
Vim Si1
Vim Si2
Ctrl Si1
Ctrl Si2
Vim Si1
Vim Si2
Ctrl Si1
Ctrl Si2
Vim Si1
Vim Si2
Vimentin
Vimentin
55 kDa
55 kDa
Actin
Actin
35 kDa
35 kDa
55 kDa
55 kDa
Tubulin
Tubulin
55 kDa
55 kDa
Keratin
Keratin
GAPDH
GAPDH
35 kDa
35 kDa
Supplementary Fig. 2
Western blotting analyses of actin, tubulin and keratin in (A) MDA-MB-231 and (B) MDA-MB-468 cells induced or not to EMT by EGF treatment, and transfected with two non-targeting siRNA (Ctrl Si1 or Ctrl Si2) or two siRNA against vimentin (Vim Si1 or Vim Si2).

## Slide 3
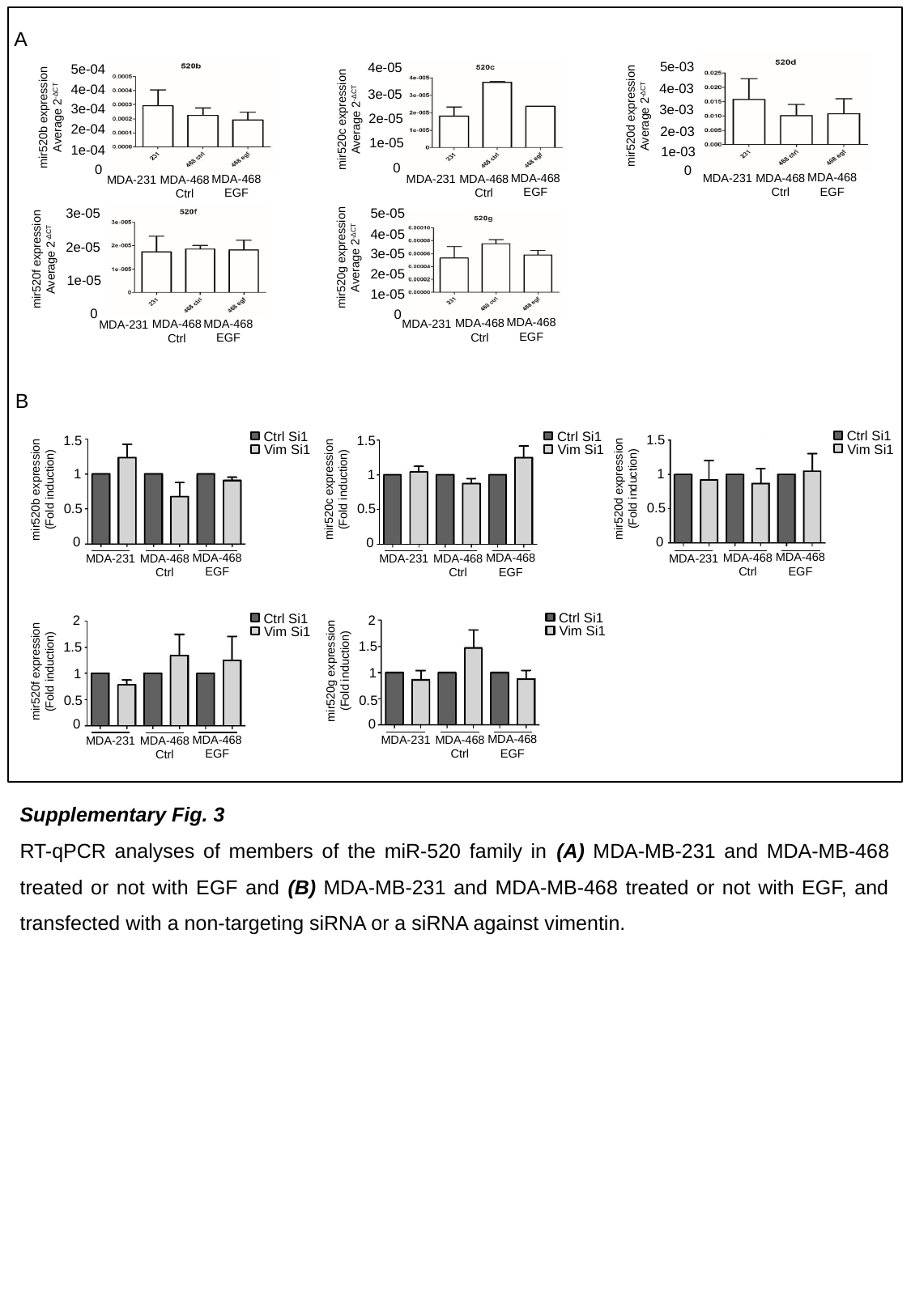

A
5e-03
4e-03
mir520d expression
Average 2-ΔCT
3e-03
2e-03
1e-03
0
MDA-468
EGF
MDA-468
Ctrl
MDA-231
5e-04
4e-04
mir520b expression
Average 2-ΔCT
3e-04
2e-04
1e-04
0
MDA-468
EGF
MDA-468
Ctrl
MDA-231
4e-05
3e-05
mir520c expression
Average 2-ΔCT
2e-05
1e-05
0
MDA-468
EGF
MDA-468
Ctrl
MDA-231
5e-05
4e-05
mir520g expression
Average 2-ΔCT
3e-05
2e-05
1e-05
0
MDA-468
EGF
MDA-468
Ctrl
MDA-231
3e-05
2e-05
mir520f expression
Average 2-ΔCT
1e-05
0
MDA-468
EGF
MDA-468
Ctrl
MDA-231
B
Ctrl Si1
Vim Si1
1.5
1
mir520d expression
(Fold induction)
0.5
0
MDA-468
EGF
MDA-468
Ctrl
MDA-231
Ctrl Si1
Vim Si1
1.5
1
mir520b expression
(Fold induction)
0.5
0
MDA-468
EGF
MDA-468
Ctrl
MDA-231
Ctrl Si1
Vim Si1
1.5
1
mir520c expression
(Fold induction)
0.5
0
MDA-468
EGF
MDA-468
Ctrl
MDA-231
Ctrl Si1
Vim Si1
1.5
mir520g expression
(Fold induction)
1
0.5
0
MDA-468
EGF
MDA-468
Ctrl
MDA-231
2
Ctrl Si1
Vim Si1
1.5
mir520f expression
(Fold induction)
1
0.5
0
MDA-468
EGF
MDA-468
Ctrl
MDA-231
2
Supplementary Fig. 3
RT-qPCR analyses of members of the miR-520 family in (A) MDA-MB-231 and MDA-MB-468 treated or not with EGF and (B) MDA-MB-231 and MDA-MB-468 treated or not with EGF, and transfected with a non-targeting siRNA or a siRNA against vimentin.
