# Supplementary material for: Vimentin prevents a miR-dependent negative regulation of tissue factor mRNA during epithelial–mesenchymal transitions and facilitates early metastasis
Source: Oncogene. 2020 Mar 10;39(18):3680–92. doi: 10.1038/s41388-020-1244-1 (PMC7190572; doi:10.1038/s41388-020-1244-1)
Supplement: Supplementary file 2 — Supplementary Tables [file 41388_2020_1244_MOESM2_ESM.docx]

**Supplementary Table 1: Mean values of FACS experiments**

| **Cell lines** |  | **Mean values** |
| --- | --- | --- |
| MDA-MB-231 | Ctrl Si1  Ctrl Si2  Vim Si1  Vim Si2 | 5866  5420  1654  3104 |
| MDA-MB-468 EGF | Ctrl Si1  Ctrl Si2  Vim Si1  Vim Si2 | 2147  1479  1099  1318 |
| A549 TGF-β1 | Ctrl Si1  Ctrl Si2  Vim Si1  Vim Si2 | 1864  1554  871  1089 |
| PMC42-LA EGF | Ctrl Si1  Ctrl Si2  Vim Si1  Vim Si2 | 191  180  71.4  133 |
| Fibroblasts | Ctrl Si1  Vim Si1 | 80.1  58.3 |

**Supplementary Table 2: List of primers used**

| **Gene** | **Primer** | **Sequence** |
| --- | --- | --- |
| hTF | Forward  Reverse | 5’ CAGACAGCCCGGTAGAGTGT 3’  5’ CCACAGCTCCAATGATGTAGAA 3’ |
| hVimentin | Forward  Reverse | 5’ GCGTGACGTACGTCAGCAATATGA 3’  5’ GTTCCAGGGACTCATTGGTTCCTT 3’ |
| 18S | Forward  Reverse | 5’ CTTCCACAGGAGGCCTACAC 3’  5’ CGCAAAATATGCTGGAACTTT 3’ |
| TBP | Forward  Reverse | 5’ GACTCCCATGACCCCCAT 3’  5’ CAACCAAGATTCACTGTGGATAC 3’ |
| hGAPDH | Forward  Reverse | 5’ accaggtggtctcctctgac 3’  5’ tgctgtagccaaattcgttg 3’ |
| hGAPDH (used as external primers to perform RT-nested qPCR) | Forward  Reverse | 5’ TGCCGTCTAGAAAAACCTGCCAAA 3’  5’ CTCTCTTCCTCTTGTGCTCTTGCT 3’ |
| mGAPDH | Forward  Reverse | 5’ TGTCCGTCGTGGATCTGAC 3’  5’ GAGTTGCTGTTGAAGTCGCA 3’ |

**Supplementary Table 3: List of antibodies used in different applications**

| **Application** | **Antibody** | **Clone** | **Source** | **Catalog numbers** |
| --- | --- | --- | --- | --- |
| **Western blotting/ IP** | Mouse anti-human  tissue factor | VD8 | Sekisui Diagnostics | ADG4508 |
|  | Mouse anti-human vimentin | V9 | Dako | M0725 |
|  | Rabbit anti-human  cytokeratin | Polyclonal | Abcam | Ab59400 |
|  | Mouse anti-tubulin | 2-28-33 | Sigma Aldrich | T5293 |
|  | Rabbit anti-actin | Polyclonal | Sigma  Aldrich | A2066 |
|  | Mouse anti-human GAPDH | 6C5 | Merck Millipore | MAB374 |
|  | Mouse IgG negative control | DAK-G01 | Dako | X0931 |
|  | Goat anti-rabbit/HRP | Polyclonal | Cell Signalling | 7074 |
|  | Horse anti-mouse/HRP | Polyclonal | Cell Signalling | 7076 |
| **Flow cytometry** | FITC-conjugated mouse anti-human tissue factor | VD8 | Sekisui Diagnostics | 4508CJ |
|  | FITC-conjugated mouse IgG1 K isotype control | P3.6.2.8.1 | eBioscience | 11-4714 |
| **Immunofluorescent staining of the mouse lung section** | Rabbit anti-mouse  Von Willebrand Factor | Polyclonal | Dako | A0082 |
|  | Rabbit anti-hKi67 | Polyclonal | Abcam | ab833 |
|  | Alexa fluor 488-conjugated donkey anti rabbit | Polyclonal | Invitrogen | A21206 |
|  | Alexa fluor 555-conjugated donkey anti mouse | Polyclonal | Invitrogen | A31570 |
|  | Rabbit anti hTF | E9M6T | Cell Signaling | 97438 |

**Supplementary Table 4: List of siRNA used**

| **siRNA** | **Sequence** |
| --- | --- |
| Vim siRNA1  Vim siRNA2 | 5’-GAAUGGUACAAAUCCAAGU-3’  5’-GCCATCAACACCGAGTTCA-3’ |
| Ctrl siRNA1  Ctrl siRNA2 | 5’-GAUACUAUCUAGUCUAGAC-3’  5’-GUCUAGACUAGAUAGUAUC-3’ |

**Supplementary Table 5: List of TSB used**

| **TSB** | **Sequence** |
| --- | --- |
| TSB ctrl | 5’ T*A*A*C*A*C*G*T*C*T*A*T*A*C*G*C*C*C*A 3’ |
| TSB 1 | 5’ C*A*A*A*G*T*G*A*C*T*A*A*T*G*C*T*G*A*T*G 3’ |

**Supplementary Table 6: List of the generated mutations on the TF 3’-UTR**

| **Number** |  | **Sequence** |
| --- | --- | --- |
| M1 | Wild type  Mutation | 5’ AGTCACTTT 3’  5’ AGTAGATTT 3’ |
| M2 | Wild type  Mutation | 5’ CATGGCA 3’  5’ GTACCGT 3’ |
| M3 | Wild type  Mutation | 5’ TTTTGCACA 3’ 5’ TTTGTAACA 3’ |
| M4 | Wild type  Mutation | 5’ CACTTT 3’ 5’ TACCTC 3’ |
| M5 | Wild type  Mutation | 5’ ACTGA 3’ 5’ TGACT 3’ |
| M6 | Wild type  Mutation | 5’ TTGCACTGTGA 3’ 5’ AACGTGACACT 3’ |
| M7 | Wild type  Mutation | 5’ GCACCTT 3’ 5’ CGTGGGG 3’ |

**Supplementary Table 7: List of primers used for miRNA identification**

| **Target miRNA** | **Sequence** |
| --- | --- |
| hsa-mir-0520b-3p | 5’- GCTTCCTTTTAGAGGGAAAAA -3’ |
| hsa-mir-0520c-3p | 5’- CAAAGTGCTTCCTTTTAGAGGGTA -3’ |
| hsa-mir-0520d-3p | 5’- GCTTCTCTTTGGTGGGTAAAA -3’ |
| hsa-mir-0520f-3p | 5’- CAAGTGCTTCCTTTTAGAGGGTTA -3’ |
| hsa-mir-0520g-3p | 5’- CAAAGTGCTTCCCTTTAGAGTGT -3’ |
| SNORD44 | 5’- GCAAATGCTGACTGAACATGAA -3’ |
| SNORD48 | 5’- CTCTGAGTGTGTCGCTGATGC -3’ |
