# Supplementary material for: Vimentin prevents a miR-dependent negative regulation of tissue factor mRNA during epithelial–mesenchymal transitions and facilitates early metastasis
Source: Oncogene. 2020 Mar 10;39(18):3680–92. doi: 10.1038/s41388-020-1244-1 (PMC7190572; doi:10.1038/s41388-020-1244-1)
Supplement: Supplementary file 3 — Supplementary Material and Methods [file 41388_2020_1244_MOESM3_ESM.docx]

**Supplementary Material and methods**

### Selection of Tumor Cancer Genome Atlas (TCGA) Dataset and mRNA expressions

To obtain external validation, we selected a breast non-metastatic Tumor Cancer Genome Atlas database (TCGA–BRCA; *n* = 1051) (1) and an independent breast-metastatic cohort (PMID: 31231679, GSE124646 ; *n* = 100) (2). Characteristic cohorts are open access and available at [https://portal.gdc.cancer.gov](https://portal.gdc.cancer.gov/) and <https://www.ncbi.nlm.nih.gov/geo>. VIM (vimentin) and F3 (tissue factor) gene expressions were then extracted as the log2 value of reads per kilobase of transcript per million mapped reads (RPKM) and their linear correlation analyzed with Spearman’s test. *p*-value ≤ 0.05 were considered significant.

**Immunohistochemistry on human lung tumors**

NSCLC samples were obtained from 80 biopsies of primary non-metastatic (n=40) or metastatic (n=40) lung carcinomas from patients who underwent surgical resection or routine fiberoptic bronchoscopy between January 2018 and August 2019 at the University Hospital of Reims. Formalin-fixed and paraffin-embedded tumor samples were obtained from the Tumor Bank of the Reims University Hospital Biological Resource Collection (NO.DC-2008-374) declared at the Ministry of Health according to the French Law, for use of tissue samples for research. This study was conducted in accordance with the ethical guideline of Declaration of Helsinki (NO.DC-2008-374). Access to patient data for this retrospective non-interventional study was approved by the French national commission for personal data protection (CNIL, Comité National de l’Information et des Libertés) (NO.2049775 v 0). All NSCLC were staged according to the 8th TNM UICC/AJCC edition. Immunohistochemical staining for TF and vimentin was performed as described previously (3). The results for immunohistochemical detection were scored independently by two pathologists who had no knowledge of the clinical data, as follows: 0= no detection, 1= detection in <25% of tumour cells, 2= detection in 26-50% of tumour cells, 3= detection in >50% of tumour cells. Two-sided Fisher’s exact test were used to analyse the association between TF and vimentin.

**Acknowledgements**

The results published here are in part based upon data generated by the TCGA Research Network: [https://www.cancer.gov/tcga](https://www.cancer.gov/about-nci/organization/ccg/research/structural-genomics/tcga).

1.	Comprehensive molecular portraits of human breast tumours. Nature **2012**;490:61-70

2.	Sinn BV, Fu C, Lau R, Litton J, Tsai TH, Murthy R*, et al.* SETER/PR: a robust 18-gene predictor for sensitivity to endocrine therapy for metastatic breast cancer. NPJ breast cancer **2019**;5:16

3.	Bourcy M, Suarez-Carmona M, Lambert J, Francart ME, Schroeder H, Delierneux C*, et al.* Tissue Factor Induced by Epithelial-Mesenchymal Transition Triggers a Procoagulant State That Drives Metastasis of Circulating Tumor Cells. Cancer Res **2016**;76:4270-82
